# Pre-Clinical Assessment of ^177^Lu-Labeled Trastuzumab Targeting HER2 for Treatment and Management of Cancer Patients with Disseminated Intraperitoneal Disease

**DOI:** 10.3390/ph5010001

**Published:** 2011-12-22

**Authors:** Geoffrey L. Ray, Kwamena E. Baidoo, Lanea M. M. Keller, Paul S. Albert, Martin W. Brechbiel, Diane E. Milenic

**Affiliations:** 1 Radioimmune and Inorganic Chemistry Section, Radiation Oncology Branch, Center for Cancer Research, National Cancer Institute, National Institutes of Health, Bethesda, MD 20892, USA; Email: x_ray82@hotmail.com (G.L.R.); baidook@mail.nih.gov (K.E.B.); lkeller8@hotmail.com (L.M.M.K.); martinwb@mail.nih.gov (M.W.B.); 2 Biostatistics and Bioinformatics Branch, Division of Epidemiology, Statistics & Prevention, *Eunice Kennedy Shriver* National Institute of Child Health and Human Development, Bethesda, MD 20892, USA; Email: albertp@mail.nih.gov (P.S.A.)

**Keywords:** ^177^Lutetium, radioimmunotherapy, trastuzumab, HER2, intraperitoneal disease

## Abstract

Studies from this laboratory have demonstrated the potential of targeting HER2 for therapeutic and imaging applications with medically relevant radionuclides. To expand the repertoire of trastuzumab as a radioimmunoconjugate (RIC) vector, use of ^177^Lu was investigated. The combination of a 6.7 d half-life, lower energy β^−^-emissions (500 keV max; 130 keV ave), and an imagable γ-emission make ^177^Lu an attractive candidate for radioimmunotherapy (RIT) regimens for treatment of larger tumor burdens not possible with α-particle radiation. Radiolabeling trastuzumab-CHX-A″-DTPA with ^177^Lu was efficient with a specific binding of 60.8 ± 6.8% with HER2 positive SKOV-3 cells. Direct quantitation of tumor targeting and normal tissue uptake was performed with athymic mice bearing subcutaneous and intraperitoneal LS-174T xenografts; a peak tumor %ID/g of 24.70 ± 10.29 (96 h) and 31.70 ± 16.20 (72 h), respectively, was obtained. Normal tissue uptake of the RIC was minimal. Tumor targeting was also demonstrated by γ-scintigraphy. A therapy study administeringescalating doses of ^177^Lu-trastuzumab to mice bearing three day LS-174T i.p. xenografts established the effective therapeutic dose of i.p. administered ^177^Lu-trastuzumab at 375 μCi with a median survival of 124.5 d while a median survival of 10 d was noted for the control (untreated) group. In conclusion, trastuzumab radiolabeled with ^177^Lu has potential for treatment of disseminated, HER2 positive, peritoneal disease.

## 1. Introduction

Human epidermal growth factor (EGF) receptor 2 (HER2 or Erb2/neu) is over-expressed in cancers such as breast, ovarian, pancreas, colorectal and others [1,2,3]. Trastuzumab (Herceptin™), a humanized monoclonal antibody (mAb) specific for the HER2 receptor, is approved for the treatment of metastatic breast cancer and has become a standard of care for this patient population [4]. Clinically, HER2 expression is detected using immunohistochemistry (IHC) or by fluorescence *in situ* hybridization (FISH). A scoring system from 0 to 3+ is employed for IHC, wherein 0 or 1+ is negative, 2+ requires FISH confirmation of gene amplification, and 3+ is positive [5,6]. Patients that score 2+ or 3+ by IHC or FISH qualify for trastuzumab therapy.

Use of mAbs as targeting vectors to selectively deliver a lethal dose of radiation to cancer cells through either a β^−^- or α-emitting radionuclide has been extensively investigated for cancer therapies [7]. Trastuzumab has proven to be an effective vehicle for targeting HER2 positive tumors to deliver therapeutic doses of radiation [8,9,10,11,12,13,14,15,16,17]. To date, studies in this laboratory have focused on and have demonstrated the therapeutic potential of trastuzumab as a delivery vehicle for α-particle radiation (using ^212^Pb and ^213^Bi) for the treatment of disseminated intraperitoneal disease [8,11,12,13,14]. The discrete energy emissions of α-particle decays (4–9 MeV) are directly deposited over a short distance (40–100 µm), resulting in high linear energy transfer. The shorter path may also have an advantage of limiting toxicity to adjacent normal tissue. As such, α-emitting radionuclides are hypothesized to be ideal for specific treatment of single cell disease, smaller tumors, low tumor burden, disseminated disease, and micrometastatic disease. Unfortunately, such radionuclides may not be effective in the presence of greater disease burden. In fact, targeted α-radiation therapy with ^213^Bi-trastuzumab was shown to be ineffective against a larger tumor burden in the peritoneum [8]. For this reason and purpose, β^−^-emitting radionuclides are of interest.

The lanthanide radiometal ^177^Lu has been identified as having favorable characteristics for RIT applications that include a path length suitable for the treatment of micrometastases and small tumors <1 cm while limiting irradiation of normal tissues [7,18]. Lutetium-177 possesses a physical half-life of 6.7 d with β^−^-emissions (E_ave_ = 133 KeV) that penetrate soft tissue 0.2 to 0.3 mm. Lutetium-177 also emits two relatively low-abundance, low-energy γ-rays (113 and 208 KeV) that are suitable for imaging with a γ-camera, while posing a lower radiation hazard to health care personnel as compared to ^131^I [18,19,20]. By virtue of the properties just listed, ^177^Lu RIT has several advantages over other β^−^-emitting radionuclides such as ^131^I, ^90^Y and ^186^Re. For example, the spectrum of decay energy of ^90^Y is only deposited in tumors with diameters of 1 cm or greater [21]. The lower energy β^−^-emissions of ^177^Lu as compared to ^90^Y translate to a smaller average cell killing diameter (12 *vs.* 150–200 µm) which may then translate to a lower risk of normal tissue toxicity. Additionally, the longer half-life of ^177^Lu (6.7 d) more appropriately matches with the biological half-life of intact mAbs (6–26 d) [22,23]. Finally, ^90^Y is a pure β^−^-emitter, thus the performance of dosimetric calculations, or the monitoring of patients and tumor response during therapy would require imaging with another radionuclide such as ^86^Y. Zirconium-89 has been proposed for this purpose which presents all of the problems associated with use of a different element as well as a different chelate antibody conjugate [24].

Prior studies from this laboratory demonstrated the potential of trastuzumab targeting of HER2 for therapeutic and imaging applications when labeled with a medically relevant radionuclide [8,11,12,13,14,25]. Translation of these studies to clinical settings would greatly expand the patient population that would benefit from treatment with trastuzumab conjugates. The aim of the present study was to develop, characterize and validate the application of ^177^Lu-trastuzumab for future clinical use with an ultimate goal of combining α- and β^−^-radiation in a treatment regimen for the management of intraperitoneal disease. The *in vitro* and *in vivo* characterization of ^177^Lu-CHX-A″-trastuzumab are presented in this report.

## 2. Experimental

### 2.1. mAb Conjugation and Radiolabeling

Trastuzumab was conjugated with the bifunctional acyclic CHX-A″-DTPA chelate by established methods using a 10-fold molar excess of ligand to trastuzumab [8,26]. The final concentration of the trastuzumab was quantitated by the method of Lowry and the average number of chelates per mAb determined by spectrophotometric assay based on the titration of the Y(III)-Arsenazo(III) complex for acyclic ligands [27,28]. Lutetium-177 (110 mCi) in the chloride form (PerkinElmer, Shelton, CT, USA) was dissolved in 100 μL of 0.1 N HCl and the pH adjusted to pH 5.5 with 5 M NH_4_OAc buffer (pH 5.5). The immunoconjugates (0.1 mg) in 0.15 M NH_4_OAc buffer (pH 6.5–7.0) were each added to the buffered ^177^Lu and incubated for 1 h at 37 °C. The reaction was quenched by the addition of 4 μL of 0.1 M EDTA (pH 6.0). The radioimmunoconjugate (RIC) was then purified using a PD-10 desalting column (GE Healthcare, Piscataway, NJ, USA). HuIgG (MP Biomedicals, Santa Ana, CA, USA), similarly conjugated and radiolabeled served as a non-specific experimental control.

### 2.2. Cell Lines

SKOV-3, a human ovarian carcinoma cell line that expresses ~1 × 10^6^ HER2 molecules per cell, was used for *in vitro* analysis [29]. The human colon carcinoma cell line (LS-174T; kindly provided by John Greiner, LTIB, NCI) utilized for the *in vivo* studies was grown in DMEM containing 10% FetalPlex (Gemini BioProducts, Woodland, CA, USA), 1 mM glutamine and non-essential amino acids as previously described [8,30]. SKOV-3 cells were maintained in McCoy’s 5a medium supplemented with 10% FetalPlex and 1 mM non-essential amino acids. The media and remaining supplements were purchased from Lonza (Walkerville, MD, USA).

### 2.3. In Vitro Studies

#### Radioimmunoassays

Immunoreactivities of the radiolabeled preparations were assessed in a radioimmunoassay using methanol-fixed cells. Briefly, SKOV-3 cells were trypsinized, pelleted and re-suspended in PBS (pH 7.2) containing 1% BSA. Serial dilutions of radiolabeled trastuzumab (~140 to 8 nCi) were added in duplicate to cells (1 × 10^6^) in 50 µL of 1% BSA in PBS. The cells were washed with 4 mL of 1% BSA in PBS following ~18 h incubation at room temperature, pelleted at 1,000 × g for 5 min and the supernatant decanted. The pellets were counted in a γ-scintillation counter (WizardOne, PerkinElmer); percent binding was calculated for each dilution and reported as an average of the dilutions. The specificity of the radiolabeled trastuzumab was confirmed by incubating one set of cells with radiolabeled trastuzumab and an excess of unlabeled trastuzumab (10 µg).

### 2.4. In Vivo Studies

#### 2.4.1. Biodistributions

Female athymic mice (nu/nu), obtained from Charles River Laboratories (Wilmington, DE, USA) at 4–6 weeks of age, were injected either subcutaneously (s.c.) in the right flank with 2.0 × 10^6^ LS-174T cells (0.2 mL) or intraperitoneally (i.p.) with 1 × 10^8^ cells (1 mL). Mice with s.c. tumors were utilized in experiments when tumors measured 0.4–0.6 cm (maximal diameter); mice bearing i.p. tumors were utilized 4–5 d after tumor implantation. The ^177^Lu-CHX-A″-trastuzumab (7.5 μCi on ~0.3 μg) was administered either i.v. (0.2 mL) via the tail vein or i.p. (0.5 mL). Mice (n = 3–5) were sacrificed by exsanguination at 24, 48, 72, 96 and 168 h post-injection. Blood, tumor and the major organs were collected, wet-weighed and counted in a γ-scintillation counter (PerkinElmer). The percent injected dose per gram (%ID/g) was determined for each organ along with the standard deviations.

#### 2.4.2. Therapy Studies

Therapy studies were performed using 19–21 g female athymic (nu/nu) mice (Charles River Laboratories, Wilmington, DE, USA). The mice were injected i.p. with 1 × 10^8^ LS-174T cells in 1 mL of medium or PBS as previously reported [31]. Escalating doses of ^177^Lu-trastuzumab, 125–750 μCi (3–16 μg), were administered to the mice (n = 6–8) 3 d post-implantation of tumor in 0.5 mL PBS. HuIgG labeled with ^177^Lu served as a non-specific control. Bloods from 3 mice in each group (50–100 μL) were drawn the day before, 4 d post-RIT, and then weekly for 90 d, diluted to 300 μL and the platelet count determined using a AcT10 hematology analyzer (BeckmanCoulter, Fullerton, CA, USA). Animal weights were also recorded twice weekly for 3–4 weeks following RIT.

Progression of disease was observed as an extension of the abdomen, development of ascites often associated with noticeable and/or palpable nodules in the abdomen. Weight loss would occur as an acute response following treatment, or, was also associated with progression of disease. Mice were monitored and euthanized if found to be in distress, moribound, or cachectic. Mice were also euthanized when 10–15% weight loss occurred, or if bloating, or tumor nodules were apparent. All animal protocols were approved by the National Cancer Institute Animal Care and Use Committee.

#### 2.4.3. Statistical Analyses

A Cox proportional hazards model was used to test for a dose response relationship between the doses of ^177^Lu-trastuzumab and survival (time to sacrifice or natural death). Groups were compared using a log-rank test. A dose-response relationship was examined by treating the dose level as a linear covariate in the Cox model and tested whether the corresponding regression parameter was zero using a likelihood ratio test.

The maximum percent reduction in weight from the baseline was estimated for each mouse. This was calculated as the ratio of the maximum reduction in weight from baseline during the initial 4-week period divided by the baseline weight of the mouse. Boxplots were constructed for each treatment group with the median, upper and lower quartiles as well as identifying outliers shown. Differences between treatment groups were tested using a Kruskal-Walis test (non-parametric ANOVA) for comparison of multiple groups and the Wilcoxon rank sum test was applied when comparing two groups. All reported p-values correspond to two-sided tests.

#### 2.4.4. γ-Scintigraphy

γ-Scintigraphy was performed with mice bearing s.c. LS-174T tumor-bearing mice to further validate tumor targeting with ^177^Lu-CHX-A″-trastuzumab. Tumor-bearing mice (n = 4–5) were given i.v. injections of 80–100 µCi (6–7.5 µg) ^177^Lu-CHX-A″-trastuzumab in 200 µL of PBS. The mice were chemically restrained with 2.5% isoflurane (Abbott Laboratories, North Chicago, IL, USA) delivered in O_2_, using a Model 100 vaporizer (SurgiVet, Waukesha, WI, USA) at a flow rate of ~1.0 L/min. Images (100,000 counts) were acquired at 24, 48, 72, and 168 h with a large field of view (LFOV) gamma camera equipped with a pinhole collimator, using a 20% window centered on both photopeaks (173 and 247 KeV).

## 3. Results and Discussion

### 3.1. In Vitro Analysis of ^177^Lu-Trastuzumab

The experiments performed were designed to evaluate the *in vitro* and *in vivo* properties of ^177^Lu-labeled trastuzumab. Previous studies from this laboratory demonstrated the suitability of the acyclic ligand, CHX-A″-DTPA for radiolabeling of mAb with ^177^Lu [32]. Modification of trastuzumab with this ligand, as previously described, was routine and resulted in a final immunoconjugate with a chelate to protein ratio of 1.7 [25]. Radiolabeling the CHX-A″-trastuzumab with ^177^Lu was efficient resulting in a specific activity of 37.7 ± 10.1 mCi/mg. The SE-HPLC analysis indicated that the radioactivity was fully associated with the mAb; retention times were consistent with those of an intact immunoglobulin (7.19 ± 0.15 min) with no indication of free ^177^Lu present (data not shown). When the RIC was evaluated by radioimmunoassay using HER2 positive (SKOV-3) cells, 60.8 ± 6.8% of the radioactivity was bound. Incubation of ^177^Lu-HuIgG with SKOV-3 cells resulted in a percent bound of 11.8 ± 6.9. Labeling of trastuzumab with ^177^Lu did not appear to have any adverse effect on the reactivity of the mAb, a result that is consistent with what has been reported for other mAbs being considered for use with this radionuclide [18,19,20,33,34,35,20,33].

### 3.2. Direct Quantitation of ^177^Lu-Trastuzumab Tumor Targeting and Normal Organ Distribution

Next, the ability of trastuzumab to target tumor when radiolabeled with ^177^Lu which was examined in both s.c. and i.p. colon carcinoma (LS-174T) tumor models. Athymic mice bearing s.c. LS-174T xenografts (n = 4–5) were injected (i.v.) with ~7.5 μCi of ^177^Lu-trastuzumab to define tumor targeting and normal organ distribution of the RIC. Blood, tumor and normal organs were harvested at 24, 48, 72, 96 and 168 h for analysis. As presented in Table 1, tumor targeting of ^177^Lu-trastuzumab was observed with a peak tumor %ID/g of 24.70 ± 10.29 at 96 h. Of the normal organs, the highest %ID/g was observed in the blood (13.72 ± 0.79) at 48 h which then decreased to 3.85 ± 1.50 by 168 h. The next highest %ID/g was observed in the liver with a value of 7.80 ± 2.52 at 24 h. Among the remainder of the normal organs, the lungs presented with the next highest %ID/g (6.57 ± 0.34) at 48 h, otherwise, %ID/g values were less than 6. These results are comparable to what has been reported from this laboratory with ^111^In-trastuzumab, in a s.c. tumor [25]. Normal organ uptake was also similar to the reported data with the exception of the blood %ID/g which was lower for the ^177^Lu- than the ^111^In-labeled trastuzumab.

**Table 1:** *In vio* distribution of i.v. injected ^177^Lu-RICs in athymic mice bearing s.c. LS-174T tumor xenografts.

		Time (h)				
RIC	Tissue	24	48	72	96	168
Trastuzumab	Blood	12.68 ± 3.08	13.72 ± 0.79	9.74 ± 2.67	10.06 ± 2.75	3.85 ± 1.50
	Tumor	16.24 ± 4.18	19.08 ± 3.23	21.75 ± 11.66	24.70 ± 10.29	19.59 ± 9.84
	Liver	7.80 ± 2.52	5.72 ± 1.57	6.39 ± 1.40	6.70 ± 1.71	5.18 ± 2.87
	Spleen	5.63 ± 1.45	3.75 ± 2.33	6.38 ± 2.64	5.69 ± 2.12	4.11 ± 2.12
	Kidney	5.33 ± 0.46	3.87 ± 2.38	4.47 ± 1.48	4.24 ± 0.62	2.54 ± 0.48
	Lung	6.51 ± 1.66	6.57 ± 0.34	5.42 ± 1.28	3.98 ± 1.75	2.10 ± 0.81
	Heart	5.06 ± 1.50	4.87 ± 0.53	3.49 ± 1.05	2.83 ± 0.81	1.33 ± 0.49
	Femur	1.67 ± 0.61	1.81 ± 0.44	1.58 ± 0.28	1.55 ± 0.49	1.05 ± 0.36
HuIgG	Blood	15.99 ± 1.99	14.81 ± 1.14	14.68 ± 2.59	11.20 ± 2.02	11.30 ± 1.59
	Tumor	7.51 ± 1.98	7.41 ± 0.73	5.70 ± 0.96	5.06 ± 1.89	7.68 ± 2.16
	Liver	6.33 ± 1.61	6.21 ± 0.77	7.35 ± 0.74	6.14 ± 2.43	5.32 ± 0.82
	Spleen	5.63 ± 0.86	5.74 ± 1.39	5.83 ± 1.08	9.88 ± 8.79	6.56 ± 1.56
	Kidney	6.00 ± 0.87	6.04 ± 0.21	5.86 ± 0.83	3.84 ± 1.72	4.75 ± 0.78
	Lung	7.59 ± 0.56	7.09 ± 0.22	6.22 ± 2.19	4.88 ± 1.23	5.58 ± 1.24
	Heart	6.40 ± 1.09	5.06 ± 0.78	4.69 ± 0.87	3.73 ± 0.53	3.56 ± 0.58
	Femur	2.09 ± 0.16	1.98 ± 0.25	1.41 ± 0.69	2.19 ± 1.16	1.59 ± 0.35

The values presented are the average percentage injected dose per gram with the standard deviations.

The distribution of ^177^Lu-HuIgG was also evaluated in the same s.c. tumor model for consideration as a non-specific control for radioimmunotherapy studies. When ^177^Lu-HuIgG is administered by i.v. injection, the highest %ID/g obtained was in the blood at 24 h (15.99 ± 1.99) which decreased only to 11.30 ± 1.59 by the end of the 168 h study (Table 1). In contrast, the tumor %ID/g is only 7.51 ± 1.98 at 24 h and appears to be static since at 168 h the %ID/g is 7.68 ± 2.16. In fact, the tumor %ID/g values do not appear to differ greatly from the normal organs. The spleen is the exception to this with a value of 9.88 ± 8.79 at 96 h post-injection of the ^177^Lu-HuIgG.

Salouti *et al.* [36] reported on the effective targeting of s.c. tumor xenografts (breast carcinoma) in mice by ^177^Lu-trastuzumab. In this situation, the trastuzumab had been conjugated with the macrocyclic DOTA ligand via an active ester moiety. In this study, the blood %ID/g, is appreciably higher at 24 h than what is reported herein. The bone uptake is also higher and shows an increase with time. This latter observation would suggest that the RIC preparation is either being degraded or the ^177^Lu is dissociating from the chelate. In fact, earlier studies from the same group characterizing their ^177^Lu-DOTA-trastuzumab preparation indicated the preparation was unstable in serum or PBS. The preparation that was characterized is suspect since free ^177^Lu is evident [10].

### 3.3. Evaluation of ^177^Lu-Trastuzumab for Targeting Intraperitoneal Tumor Burden

The utility of targeting of disseminated peritoneal disease with ^177^Lu-trastuzumab was confirmed in the i.p. model; HuIgG was evaluated as the non-specific control for the planned therapy study. Mice bearing 5 d peritoneal LS-174T xenografts were injected i.p. with ^177^Lu-trastuzumab or ^177^Lu-HuIgG (~7.5 μCi) with tumor, blood and normal tissues collected for analysis at 24 and 72 h. Tumor tissue was macroscopically identified, excised and obvious adherent tissue removed. Again, tumor targeting was obtained with a %ID/g of 31.44 ± 11.52 at 24 h and 31.70 ± 16.20 at 72 h (Table 2). Tumor masses, ranging from 30–336 mg (123 ± 96 mg), were harvested from multiple sites throughout the peritoneum. Surprisingly, the normal organs presented with %ID/g similar to what was obtained in the mice bearing s.c. tumor xenografts; the spleen having the highest %ID/g of the normal organs at 72 h (10.63 ± 6.37). Targeting of the i.p. tumor xenografts with the ^177^Lu-trastuzumab was also obtained albeit not at the same level as reported for ^111^In-trastuzumab. Specificity of tumor targeting was confirmed by the low level of tumor uptake of ^177^Lu-HuIgG in both of these tumor models.

**Table 2:** *In vivo* distribution of i.p. injected ^177^Lu-RICs in athymic mice bearing i.p. LS-174T tumor xenografts.

		^177^Lu-Trastuzumab			^177^Lu-HuIgG	
Tissue		24 h	72 h		24 h	72 h
Blood		10.47 ± 9.19	10.15 ± 6.27		16.8 ± 2.60	5.13 ± 3.17
Tumor		13.44 ± 11.52	31.70 ± 16.20		5.84 ± 1.27	5.52 ± 4.92
Liver		4.83 ± 3.63	6.11 ± 3.33		6.76 ± 1.01	2.95 ± 2.06
Spleen		6.81 ± 5.66	10.63 ± 6.37		6.67 ± 1.17	4.22 ± 2.42
Kidney		4.46 ± 3.45	5.05 ± 1.69		7.07 ± 1.25	2.46 ± 1.29
Lungs		4.81 ± 3.84	4.30 ± 2.17		7.48 ± 1.70	2.28 ± 1.24
Heart		3.60 ± 2.97	3.59 ± 1.80		6.01 ± 1.23	1.70 ± 1.03
Femur		1.96 ± 1.58	2.54 ± 1.05		2.72 ± 0.68	1.19 ± 0.62

The values presented are the average percentage injected dose per gram with the standard deviations.

Overall, whether ^177^Lu-CHX’A″-trastuzumab was administered i.v. (Table 1) or i.p. (Table 2), the RIC demonstrated targeting of tumor tissue and maintained stability. These results suggest positive *in vivo* tumor targeting for the ^177^Lu radiolabeled trastuzumab with low uptake of the RIC agent by normal tissues.

### 3.4. Validation of ^177^Lu-Trastuzumab as a Radioimmunotherapeutic and Determination of an Effective Therapeutic Dose

Having determined that ^177^Lu-trastuzumab was suitable for pursuing as a RIT agent, a study was conducted to determine the effective therapeutic dose of the RIC in mice bearing i.p. tumor (LS-174T) xenografts. Athymic mice (n = 6–9) bearing 3-day LS-174T (i.p.) xenografts were treated with a single i.p. injection of ^177^Lu-trastuzumab with the doses ranging from 125 to 750 μCi, to assess the therapeutic efficacy of this RIC. One group of mice received PBS while other groups received 125, 375 and 500 μCi of ^177^Lu-HuIgG. Mice that received only PBS had a median survival (MS) of 10 d (Table 3). The MS in the groups that received 125, 250 and 375 μCi of ^177^Lu-trastuzumab was 46, 91 and 124.5 d, respectively, suggesting a dose-dependent response to the therapy, although differences between the five doses of ^177^Lu-trastuzumab were not statistically significant (P = 0.27). The 375 μCi dose resulted in a 12-fold increase in the median survival. Therapeutic benefit appears to be lost at the 500 μCi dose with a MS of 69.5 d and the MS of 17 d at 750 μCi suggests that the maximum tolerated dose lies between the 375 and 750 μCi doses. The non-specific control, ^177^Lu-HuIgG, was evaluated at 125, 375 and 750 μCi. The MS at these doses was 14, 31 and 16 d respectively. These differences were not statistically significant (P = 0.08).

**Table 3:** Median survival of athymic mice (n = 6–8) bearing i.p. LS-174T tumor xenografts following i.p. ^177^Lu-trastuzumab RIT.

Dose (μCi)	Treatment				
	None	Trastuzumab		HuIgG	
	Median Survival(d)	Median Survival(d)	Therapeutic Index	Median Survival(d)	Therapeutic Index
0	10				
125		46	4.6	14	1.4
250		91	9.1	---	---
375		124.5	12.45	31	3.1
500		69.5	6.95	---	---
750		17	1.7	16	1.6

Therapeutic Index is the median survival of the treatment group divided by the median of survival of the group without any treatment.

**Figure 1:**
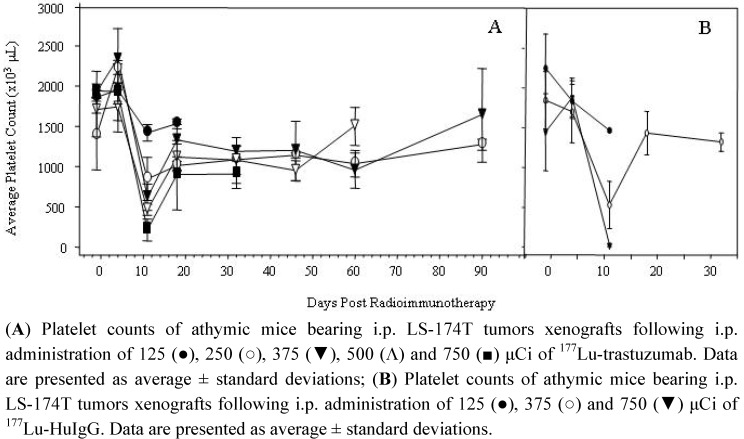
Effect of ^177^Lu-RIT on platelet counts of athymic mice bearing i.p. LS-174T tumor xenografts.

Toxicity of the ^177^Lu-RIT was assessed by monitoring platelet counts and animal weights. Bloods were drawn the day before, 4 d post-RIT and then weekly for 90 d to evaluate the effect of the ^177^Lu-RIT on platelet count and to provide additional information on the appropriate dose of ^177^Lu for future therapy studies. As depicted in Figure 1, the nadir for all of the dose treatments occurred at 11 d; the platelet counts were 76, 61, 32, 27 and 11% of the pre-treatment counts for the 125, 250, 375, 500 and 750 μCi doses of ^177^Lu-trastuzumab, respectively (Figure 1A). At 750 μCi, the platelet count reached a low of 212 k/μL from which the animals failed to recover. At the subsequent time point, one week later, platelet counts were 88, 73, 69, 65 and 46% of the original counts. When the last blood was drawn for the 750 μCi group at 32 d, the blood count was 900 k/μL, 47% of the counts before initiation of the RIT. Meanwhile, at 90 d, the platelet counts for the groups receiving 250 and 375 μCi were 92 and 100% of the initial counts. A more pronounced impact on platelet count was observed in the groups injected with ^177^Lu-HuIgG (Figure 1B). At 11 d, the platelet counts at the 125, 375 and 750 μCi doses were 1,464, 528 and 20 k/μL; 65, 28 and 1.3% of the pre-RIT counts. In these sets of animals, only animals in the 375 μCi dose group survived long enough for an additional blood draw and platelet count; at 32 d the platelet count was 71% of the pre-treatment counts.

**Figure 2:**
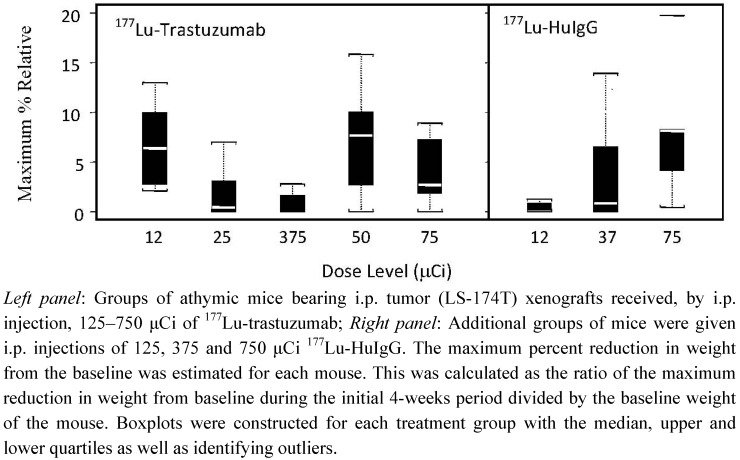
Effect on body weight of athymic mice bearing i.p. LS-174T tumor xenografts receiving increasing doses of ^177^Lu-RIT.

Animal weights were monitored for 21–28 d following RIT. The maximum percent reduction in weight relating to the five dose levels studied is depicted in Figure 2. There is a clear effect at the 500 and 750 μCi doses of ^177^Lu-trastuzumab on animal weight consistent with the survival data. A test of the differences between the groups was statistically significant with the mildest toxicity at the 375 μCi group (P = 0.019). There is a dose dependent effect on the weights of the mice that received the non-specific control (P = 0.010). Furthermore, at the 375 μCi dose, the ^177^Lu-HuIgG has a greater impact on animal weights, a greater reduction than the ^177^Lu-trastuzumab.

The higher doses of 500 and 750 μCi were associated with toxicity, determined by weight loss and platelet count. Not only did the group receiving 375 μCi of ^177^Lu-trastuzumab experience the lowest weight loss, but the platelet counts recovered to 86% of their pre-RIT level 90 d post-treatment. Again, specificity of this therapeutic effect was demonstrated by comparison of the response to ^177^Lu-HuIgG. There is a modest non-specific radiation effect observed with the 375 μCi dose of ^177^Lu-HuIgG; however, it represents only a 3.1-fold increase in the median survival in this group of mice.

The effective therapeutic dose chosen for ^177^Lu-trastuzumab in these studies is comparable to what was chosen for the treatment of s.c. prostate cancer xenografts with ^177^Lu-hu3S193, an mAb that recognizes the Le^Y^ antigen [33]. These studies, which used the CHX-A″-DTPA for radiolabeling the hu3S193 mAb, resulted in an increase in MS from 36 d (mock treated) to 133 d with a dose of 350 μCi of ^177^Lu-hu3S193.

Targeting of HER2 in SKOV-3 xenografts has also been reported using pertuzumabas the targeting vehicle for^ 177^Lu [33,34,35]. Tumor targeting studies with this RIC were akin to those reported here. A therapy study in which 189 and 135 μCi of ^177^Lu-pertuzumab was administered to tumor bearing mice resulted in a delay in tumor growth and an apparent increase in the MS.

### 3.5. Validation of Tumor Targeting with ^177^Lu-Trastuzumab by Planar γ-Scintigraphy

**Figure 3:**
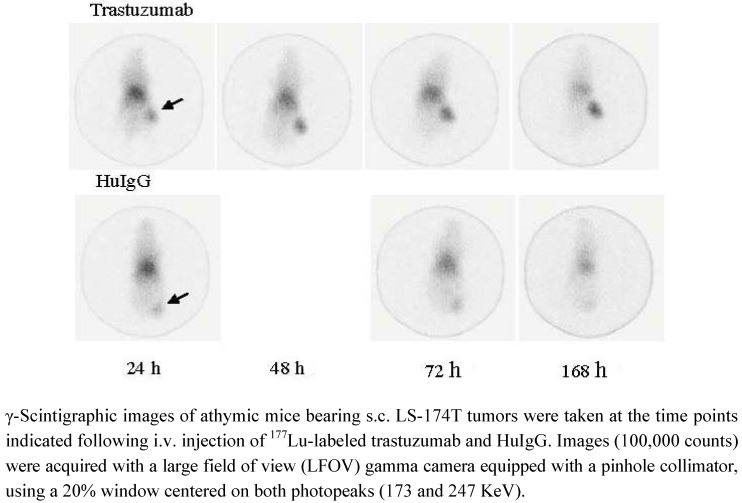
γ-Scintigraphy of ^177^Lu-trastuzumab targeting LS-174T s.c. tumors.

The images taken by γ-scintigraphy shown here also highlight advantages of ^177^Lu (over ^90^Y) which possesses two relatively low-abundance, low-energy imagable γ-rays (113 and 208 KeV). This property permits monitoring of patients during RIT and for dosimetry calculations to be performed. As depicted in Figure 3, localization of the RIC in the s.c. LS-174T xenograft on the rear flank of the mouse is clearly evident by γ-scintigraphy at 24 h with some activity present in the heart, lungs and liver. This activity was found to persist in the tumor through the 168 h study while the background signal (heart, lungs and liver) decreased. Specificity of the ^177^Lu-trastuzumab is confirmed by the absence of tumor uptake of ^177^Lu-HuIgG. Similar results have been reported for pre-clinical studies by other investigators when CHX-A″-DTPA was employed for the radiolabeling of the mAb [33,35]. Images of poorer quality resulted when NHS-DOTA was used [36,37]. In two studies, with two different mAbs (trastuzumab and PR81) and tumor models, a higher level of background signal is evident in the γ-scintigraphic images presented. The level of radioactivity attributable to the heart, lung and liver (blood pool) does not appear to clear over 4 or 7 d in either of these two studies. Furthermore, the radioactivity in the tumor, where ^177^Lu-DOTA-trastuzumab is targeting a breast cancer xenograft, appears to diminish with time, not persisting in the tumor [9]. As mentioned earlier, this may be a result of dissociation of the ^177^Lu from the trastuzumab, the injection of a suboptimal preparation of the RIC, or a combination thereof.

## 4. Conclusions

Numerous pre-clinical and clinical studies have reported on the potential of targeting HER2 for imaging and therapeutic applications using trastuzumab labeled with medically relevant radionuclides [8,9,10,11,12,13,14,15,16,17,25,38,39,40,17,25,38]. Extensive studies conducted in this laboratory with trastuzumab radiolabeled with ^111^In, ^86^Y and ^213^Bi have utilized the CHX-A″-DTPA chelate [8,25]. This laboratory has focused on optimizing the use of the α-particle emitting radionuclides, ^213^Bi and ^212^Pb, for the treatment of disseminated peritoneal disease [8,11,12,13,14]. Trastuzumab labeled with ^213^Bi was found effective for the therapy of disseminated peritoneal disease, however, as is postulated for targeted α-particle radiation, it was not effective against a larger tumor burden [8]. The short path length of α-radiation, however, is not effective for larger tumor masses, hence the continued interest in the medically relevant radioisotopes with β^−^ emissions [8,41]. One candidate in this category under evaluation has been ^177^Lu which has several attractive properties. The focus of the studies reported herein was to evaluate the therapeutic potential of trastuzumab labeled ^177^Lu with the goal of developing a therapeutic regimen ultimately combining α- and β^−^-radiation for the treatment of disseminated peritoneal disease. The ultimate goal of this combined modality is to extend the ability of RIT to manage patients with cancers presenting in the peritoneum at later stages of their disease when greater tumor burdens are present. The studies detailed above demonstrate the flexibility of trastuzumab as a RIC for both imaging and therapeutic applications for the treatment and management of a spectrum of cancers.
